# Prediction of regulatory elements in mammalian genomes using chromatin signatures

**DOI:** 10.1186/1471-2105-9-547

**Published:** 2008-12-18

**Authors:** Kyoung-Jae Won, Iouri Chepelev, Bing Ren, Wei Wang

**Affiliations:** 1 Dept of Chemistry & Biochemistry, University of California San Diego, 9500 Gilman Drive, La Jolla, CA 92093-0359, USA; 2 Ludwig Institute for Cancer Research, University of California San Diego, 9500 Gilman Drive, La Jolla, CA 92093-0653, USA

## Abstract

**Background:**

Recent genomic scale survey of epigenetic states in the mammalian genomes has shown that promoters and enhancers are correlated with distinct chromatin signatures, providing a pragmatic way for systematic mapping of these regulatory elements in the genome. With rapid accumulation of chromatin modification profiles in the genome of various organisms and cell types, this chromatin based approach promises to uncover many new regulatory elements, but computational methods to effectively extract information from these datasets are still limited.

**Results:**

We present here a supervised learning method to predict promoters and enhancers based on their unique chromatin modification signatures. We trained Hidden Markov models (HMMs) on the histone modification data for known promoters and enhancers, and then used the trained HMMs to identify promoter or enhancer like sequences in the human genome. Using a simulated annealing (SA) procedure, we searched for the most informative combination and the optimal window size of histone marks.

**Conclusion:**

Compared with the previous methods, the HMM method can capture the complex patterns of histone modifications particularly from the weak signals. Cross validation and scanning the ENCODE regions showed that our method outperforms the previous profile-based method in mapping promoters and enhancers. We also showed that including more histone marks can further boost the performance of our method. This observation suggests that the HMM is robust and is capable of integrating information from multiple histone marks. To further demonstrate the usefulness of our method, we applied it to analyzing genome wide ChIP-Seq data in three mouse cell lines and correctly predicted active and inactive promoters with positive predictive values of more than 80%. The software is available at .

## Background

Transcriptional regulation in eukaryotic cells requires highly orchestrated interactions between transcription factors (TFs), their co-factors, RNA polymerase and the chromatin [1,2]. Several classes of regulatory elements, including promoters, enhancers, silencer and insulators, are involved in this process. Systematic and precise mapping of these elements in the genome is essential for understanding transcriptional programs responsible for temporal and tissue specific gene expression. A high throughput experimental approach has recently been used to tackle this problem and it involves the chromatin immunoprecipitation assay followed by microarray (ChIP-chip)[3,4] or large scale sequencing (ChIP-Seq)[5-8]. Currently, this approach is still limited by the availability of antibody specifically recognizing individual TFs at different regulatory elements. Another method involves comparative genomic analysis of related genomes[9,10] and clustering of multiple sequence motifs[11-13]. This approach has been successfully applied to a number of eukaryotic genomes including yeast, Drosophila and mammal genomes (see review, for example, [14]). These methods rely on precise alignment of regulatory elements across multiple genomes which is not necessarily true for all elements, or prior knowledge of a set of cooperative TFs which is not always available.

Recently, a chromatin based regulatory element mapping approach has been proposed[15]. This approach exploits the observation that transcriptional promoters and enhancers are associated with distinct chromatin signatures. Specifically, the active promoters are characterized by tri-methylation on Lys4 in H3 (H3K4me3), while the active enhancers are associated with mono methylation of this residue and a much reduced or non-existent signal of the tri-methylation [15]. Currently, it is not yet clear what mechanisms underlie the different chromatin signatures at these two classes of *cis*-regulatory sequences, but the characteristic chromatin signatures of regulatory elements provide a pragmatic way to systematically identify these elements in the genome without prior knowledge of the underlying sequences. Compared with the other methods, there are several advantages of this chromatin-based approach. First, it requires no prior knowledge of the sequence features of the promoters or enhancers; Second, the chromatin modification profiles could be obtained for most organisms as the existing antibodies can specifically recognize the characteristic histone modifications in different species. Third, this approach does not make the assumption that promoters or enhancers are evolutionarily conserved, thereby can identify fast evolving regulatory elements in the genome.

**Figure 1:**
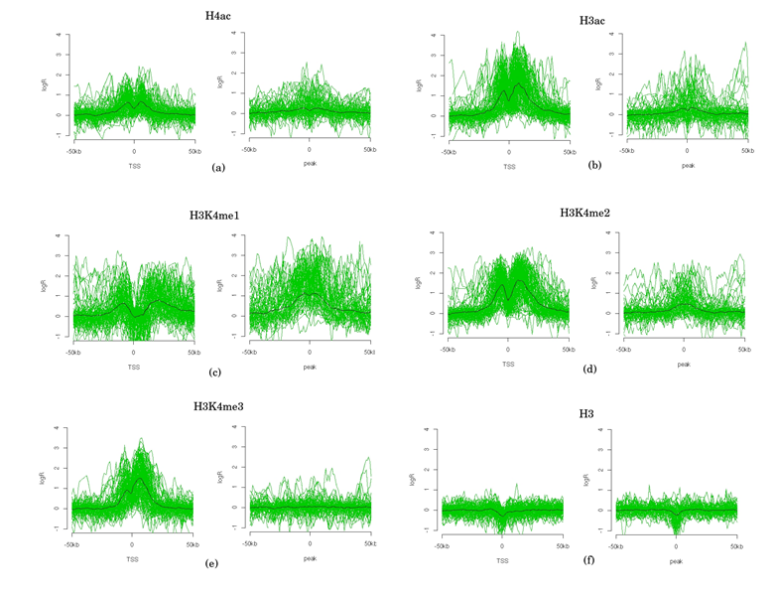
**Histone modification patterns of promoters and enhancers in untreated HeLa cells**. This figure is re-generated from Heintzman *et al *[15]. All signals of six histone marks are drawn centered on TSSs and p300 binding peaks. Average signal of histone marks of TSS and enhancers are drawn in black.

Distinct chromatin signatures at promoter and enhancers have been explored by Heintzman *et al*[15] to map promoters and enhancers. In their study, ChIP-chip analysis using high-resolution tiling array was performed to localize the core histone H3 (referred as H3) and monitor the status of five histone modification marks, i.e. H4 acetylation (H4ac), H3 acetylation (H3ac), mono-, di- and tri-methylation of Lys4 in H3(H3K4me1, H3K4me2 and H3K4me3) in HeLa cells before and after treatment with interferon-gamma (IFN*γ*). In addition, binding sites for components of transcription machinery (RNAPII and TAF1) and p300 (a transcriptional co-activator) were identified to locate active promoters and enhancers, respectively. Using these functional sites, Heintzman *et al*[15] determined characteristic chromatin modification profiles at the promoters and enhancers – promoters have both H3K4me1 and H3K4me3 marks in contrast to the prominent H3K4me1 presence at enhancers with much reduced H3K4me3 signal. Using the average profiles of the promoters and enhancers as templates, Heintzman *et al*. identified additional genomic regions sharing similar profiles and confirmed that many of the predictions indeed correspond to promoters and enhancers. Figure 1 shows the averaged profile of the histone profiles they studied. By comparing the prediction performance of all possible combinations of the six histone marks using cross-validation, they concluded that the combination of H3K4me1 and H3K4me3 best discriminated promoters from enhancers.

**Figure 2:**
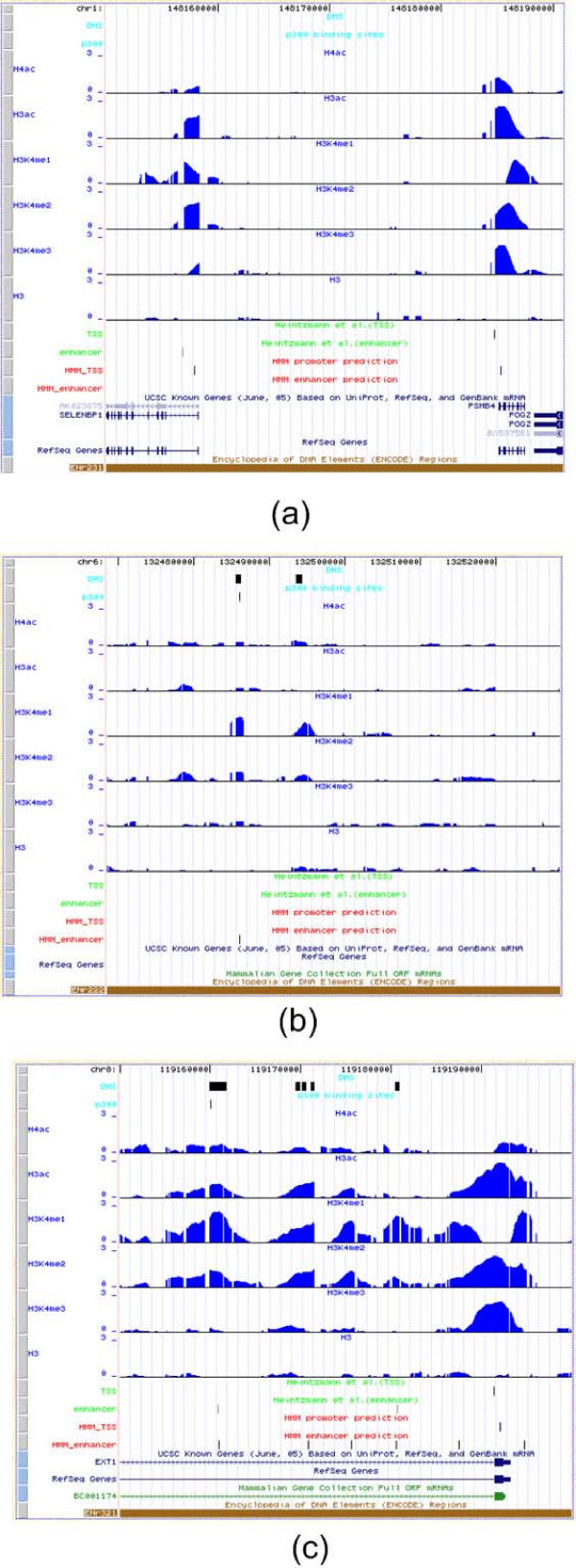
**Examples of histone modification patterns in promoters and enhancers**. (A) Promoter prediction using chromatin signature. TSS near chr1:148185131 shows a typical histone modification pattern for promoter while H3K4me3 has a relatively weak signal for the TSS near chr1:148158254. The predictions made by the profile based method of Heintzman *et al*. are labeled in green and the predictions made by the HMM developed in this study are in red. (B) Enhancer prediction using chromatin signature. A p300 binding site is shown at chr6:132486009 and overlaps with a DHS site, which is a strong evidence to support an enhancer site. (C) Enhancer prediction using chromatin signature. A DHS site near chr8:119170000 overlaps with a weak H3K4me3 signal but is not found as an enhancer by the profile-based method.

In spite of the success of this profile-based method in predicting promoters and enhancers, it is limited in two aspects. First, the optimal performance of the method involves only two histone modification marks, therefore the prediction accuracy was sensitive to the noise of measurements of these two marks. The contribution of other chromatin modifications marks to the classification method and the interdependency of the histone marks were not considered. Second, the window size of histone modification patterns (10 kb) was chosen in an arbitrary way. The larger the window size, the smaller the portion of the central regions with the strongest signal intensity. Thus, the profiles built for the promoter/enhancer may not be optimal. Figure 2(A) shows examples of histone modification patterns and annotated genes in human chromosome 1. The TSSs of these genes are well aligned with strong histone patterns of promoters. The profile-based method by Heintzman *et al*. correctly identified the promoter near chr:148185131 but not the one near chr1:148158254 because of the relatively weak H3K4me3 signal. An enhancer was also identified close to chr1:148158254 because of weak H3K4me3. In Figure 2(B) a DHS region and a p300 binding peak overlap at chr6:132486009, showing strong evidence of enhancer. Since the H3K4me3 signal is relatively stronger than a typical enhancer profile which almost has no signal, the profile based method missed this site. Another example near chr8:119170000 shows weak pattern of H3K4me3, which misleads the prediction (Figure 2(C)).

To overcome the above limitations, we developed a method coupling HMM with simulated annealing (SA) [16] (a HMM-SA procedure) to identify promoters and enhancers based on chromatin signatures. The HMM is capable of extracting more information from the chromatin modification profile signals, is less sensitive to the measurement noise of an individual histone mark, and can automatically select the most informative combination of histone marks as well as the optimal window size. In each run of SA we trained HMMs[17,18] using the 105 promoters and 73 enhancers determined by the ChIP-chip experiments on RNAPII, TAF1 and p300[15]. Inside each HMM, the histone patterns are regarded as continuous observation densities emitted from the HMM states. The number of histone patterns is the input dimension of the HMM. The optimal combination and window size of histone modifications to discriminate promoters from enhancers were searched using the HMM-SA procedure. We then used the trained HMMs to predict promoters and enhancers in the entire ENCODE regions. Below, we describe this method and the results comparing the performance of our new method with the previous method. We also demonstrated that including more histone marks can further boost the performance of our method, which is also distinct from the profile-based method. In addition, we showed the usefulness of our method on predicting the activity of promoters in the mouse genome using histone modification data generated by ChIP-Seq [8].

## Results and discussion

### Find the most informative combination and the optimal window size of histone modifications

To characterize chromatin signature of promoters and enhancers, one needs to define the histone modifications that can discriminate different regulatory elements. Since the chromatin signals from ChIP-chip analysis typically span thousands of base pairs, a small window may not fully capture the chromatin signature while a large window may include non-informative regions to deteriorate the prediction accuracy. Therefore, an optimal window size is critical in predicting promoters and enhancers using histone modification patterns. To find the most informative combination and the optimal window size, we coupled the hidden Markov model (HMM) with simulated annealing (SA) [16] (see Methods).

To compare with the profile-based method, we considered the 105 promoters and 73 enhancers determined by the ChIP-chip experiments on RNAPII, TAF1 and p300 in the Heintzman *et al*. study[15]. The datasets were divided into two equal sets, one for training and one for evaluation. The HMM-SA procedure started with a random combination of histone marks and a random window size chosen from 1, 2, 4, 6, 8, 10 and 12 kb centered on the TSSs or p300 peaks. We have conducted 100 independent simulations and collected all the final outputs of the combinations of histone marks and the window size.

We found the window size of 2 kb to be the optimal window size in 75 out of 100 simulated annealing runs. As shown in Figure 1 the strongest and the most informative signals are close to the center but 1 kb-window may be too small to capture the characteristic patterns. We also examined the occurrence of histone modification combinations in the 100 runs and compared their prediction accuracy on the evaluation set of 53 promoters and 37 enhancers (Table 1). The combination of all six marks was selected by the HMM-SA procedure 43 times, which is much higher than the other combinations. This observation is not totally unexpected because more information is included when including more histone marks. We also observed that the prediction accuracies for different combinations of multiple histone modifications are comparable with that of all six marks, which may be due to the small data set we have and the dependency of HMMs on the initial conditions. The combination of all six histone marks was chosen most often because it has higher chance to get better result in the SA test and insensitive to the choice of initial conditions. For a larger dataset for training and evaluation, the differences between the prediction accuracies of different histone mark combinations are expected to be more significant.

**Table 1:** The results of 100 HMM-SA runs.

Combination		
H4ac, H3ac, H3Kme1, H3K4me2, H3K4me3, H3	43	98.8%/94.5%

H3Kme1, H3K4me2, H3K4me3, H3	8	99.1%/93.2%

H4ac, H3ac, H3Kme1, H3K4me2, H3	6	99.1%/94.1%

H3Kme1, H3K4me2	6	99.7%/92.8%

H4ac, H3Kme1, H3K4me2, H3K4me3,	5	100%/93.5%

H3Kme1, H3K4me2, H3K4me3, H3	5	100%/93.0%

H3ac, H3Kme1, H3K4me2, H3K4me3, H3	5	99.2%/94.6%

H3Kme1, H3K4me2, H3K4me3	5	99.6%/94.6%

H4ac, H3Kme1, H3K4me2, H3	4	100.0%/93.2%

H4ac, H3ac, H3Kme1, H3K4me2, H3K4me3	3	98.1%/94.6%

H4ac, H3Kme1, H3K4me2	3	100%/94.6%

H4ac, H3ac, H3Kme1, H3K4me3, H3	2	97.2%/93.2%

H3Kme1, H3K4me2, H3	2	100.0%/93.2%

H4ac, H3K4me3	1	96.2%/91.9%

H3ac, H3Kme1, H3K4me2	1	98.1%/94.6%

H3ac, H3Kme1, H3K4me2, H3	1	100.0%/94.6%

Window Size	Number of times used	Prediction rate (promoter/enhancer)

1 K	8	99.3%/93.6%

2 K	75	99.0%/94.3%

4 K	7	99.7%/93.4%

8 K	7	99.5%/93.1%

10 K	2	100%/91.9%

12 K	1	98.1%/89.2%

Among the pair combinations, the H3K4me1 and H3K4me2 pair was chosen six times while the combination of H3K4me1 and H3K4me3 was not found by HMM-SA. Examining the histone modification patterns (Figure 1), it is obvious that H3K4me2 is more informative to locate enhancers than H3K4me3 because H3K4me2 shows stronger signal around p300 binding sites than H3K4me3. Since we did not just classify promoter against enhancer but rather we predicted promoter/enhancer against background, H3K4me2 was selected more often than H3K4me3. We next examined which single histone modification is the most informative by simply counting how many times a histone mark was included in the final combination (Table 2). Consistent with the above observation, H3K4me1 and H3K4me2 turned out to occur most often (99 and 97 times, respectively). This is not surprising because on average these two marks have the strongest signal among all six histone modifications (Figure 1). H3 was included 76 times even though its signal is relatively weak, which is surprising at the first glance. Further examination of H3 signals showed that they are consistent and well aligned (Figure 1), which makes it informative in the sense to help locate the center of promoter/enhancer.

**Table 2:** Occurrence of each histone modification in the most informative combinations found by the 100 HMM-SA runs.

H4ac	H3ac	H3K4me1	H3K4me2	H3K4me3	H3
75	61	99	97	77	76

### Cross validation shows that HMM method predicts enhancers more accurately than the profile-based method

Using the optimal combination and the window size the HMM-SA procedure found, we conducted five-fold cross-validation tests to compare the performance of the proposed method with the profile-based method[15]. There were 105 promoter and 73 enhancer profiles in our analysis (see Methods). We used three hidden states to train the HMM for promoters and enhancers separately (see Methods). In total, we performed 30 independent cross-validation tests and the averaged results are shown in Table 3. The HMM and the profiles-based method have a comparable accuracy on predicting promoters (positive predictive value (PPV = TP/(TP+FP)) = 97.87 ± 1.06% versus 96% using all six histone marks). In contrast, significant improvement over the profile-based method was indeed observed on enhancer prediction: 93.52 ± 1.83% using all the six histone marks by the HMM versus 78% using all six marks or 85% using H3K4me1 and H3K4me3 by the profile-based method. It is not surprising that the HMM using all six marks outperforms the profile-based method using only H3K4me1 and H3K4me3 because more information is included by using more marks. However, as shown by Heintzman *et al*., the profile-based method achieved the best performance using two but not all the six marks. This may explain that the HMM can capture the characteristic pattern better than using profile, particularly for enhancers that have relatively weaker signals than promoters. This is further supported by the observation that the HMM using only H3K4me1 and H3K4me3 still achieved much higher prediction accuracy on enhancers than the profile-based method (PPV = 94.06 ± 0.89% in Table 3).

**Table 3:** Comparison of cross-validation results for predicting promoters and enhancers.

Combination	Promoter PPV^a ^(standard deviation)	Enhancer PPV (standard deviation)
HMM method using 6 histone signatures^b^	97.87% (1.06)	93.52%(1.83)

HMM method using 2 histone signatures^c^	95.46% (2.82)	94.06% (0.89)

Heintzman *et al. *using 6 histone signatures	96%	78%

Heintzman *et al. *using 2 histone signatures	95%	85%

^a^Positive predictive value (PPV) = true prediction/(true prediction + wrong prediction) and standard deviation are calculated.

^b^6 histone signatures: H4ac, H3ac, H3Kme1, H3K4me2, H3K4me3, H3

^c^2 histone signatures: H3Kme1, H3K4me3

### Analysis on the trained HMM

After we validated the HMM model using cross-validations, we further examined the probability density distribution of each state in the HMM. The 3-state HMMs with no backward transition were trained on promoters and enhancers separately. This type of structures without backward transitions has been widely used in speech recognition to capture the pattern of speech [17]. The second state usually corresponds to the location of TSS or p300 peaks in this configuration. The first and the third states correspond to the upstream and downstream profiles of chromatin modifications, respectively. Figure S1 (A,B,C,D,E,F) (see Additional files 1, 2, 3, 4, 5, 6) shows the probability density of Gaussian mixtures for the three states of every chromatin mark on promoters and enhancers. It is obvious that promoters and enhances present differences in distributions of probability density, which reflects the chromatin modification patterns in these regions. For example, the probability density of H3K4me3 showed peaks in the high ChIP-chip ratio regions for promoters compared to peaks in the low ratio regions for enhancers (Figure S1(E), see Additional file 5). In addition, examining the probability density distribution of the three states in the promoters suggested that the HMM model also captured the characteristics of the chromatin modification profiles. The probability density functions of the third state for H3K4me3 were peaked around 2.5 of ChIP-chip log ratio. The second state and the first state peaked at low ChIP-chip ratios with lower probability. This is indeed a bimodal pattern with higher ChIP-chip ratios on the downstream, which is consistent with the finding in the previous study [15].

### Promoter prediction in the ENCODE regions

**Figure 3:**
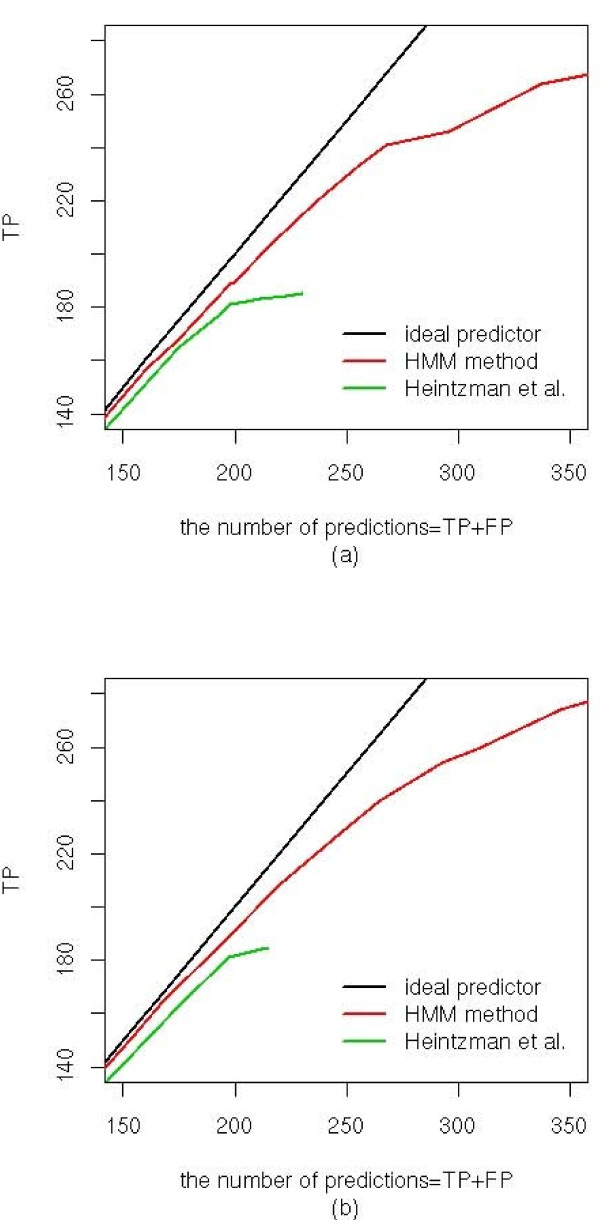
**True positives (TPs) versus the total number of promoter predictions (a) in the untreated and (b) in the treated HeLa cells**. The TF was calculated at different cutoff values of the log-odds (see Methods). Ideal predictors are shown with a black line.

We then applied our model to predicting promoters in the entire ENCODE regions of HeLa cells before and after treatment with IFN*γ*. We examined the classification performance of the HMM classifier by counting how many promoter predictions were supported by the annotated TSSs [19], CAGE tags [20] and active promoters [21]. We compared the results with the promoter predictions of Heintzman *et al*. They reported 198 and 208 TSS predictions in the untreated and IFN*γ *treated HeLa cells in the ENCODE regions [15]. Figure 3 plots the number of predictions supported by the annotated TSSs against the total number of predictions by varying the cutoff *c*_1 _from 0.4 to 3.5 (see Methods). We observed the same number of promoter predictions as the profile-based method at the cutoff (*c*_1_) of 2.205 in the untreated HeLa cells, and at *c*_1 _= 2.1 in the treated cells (Table 4). We found that 77% and 73% of predictions by the HMM and the profile based method were common for the untreated and treated cell, respectively (Table 4). The HMM method (PPV = 189/198 = 95.45%) did outperform the profile-based method (PPV = 181/198 = 91.41%). The HMM method predicted more annotated TSSs when the PPV of the two methods were similar to each other: in the untreated cell PPV = 234/256 = 91.41% and in the treated cells PPV = 247/279 = 88.50%. When we further increased the number of prediction the total number of correct predictions reached to around 270 TSSs using the HMM method, while the maximum number of the correct prediction of the profile method was 190 correct predictions (Figure 3).

**Table 4:** Comparison of PPV = TP/(TP+FP) in promoter predictions using the annotated TSS sites.

Untreated				
	Total Prediction (TP+FP)	TP	PPV	p-value

Heintzman *et al.*	198	181	91.41%	< 1.0 × 10^-16^

HMM(*c*_1 _= 2.205)	198	189	95.45%	< 1.0 × 10^-16^

HMM (*c*_1 _= 1.6)	256	234	91.41%	< 1.0 × 10^-16^

HMM (*c*_1 _= 0.5)	337	264	78.34%	< 1.0 × 10^-16^

Treated				

	Total Prediction (TP+FP)	TP	PPV	p-value

Heintzman *et al.*	207	183	88.41%	< 1.0 × 10^-16^

HMM (*c*_1 _= 2.1)	207	196	94.69%	< 1.0 × 10^-16^

HMM (*c*_1 _= 1.367)	279	247	88.50%	2.8 × 10^-3^

HMM (*c*_1 _= 0.5)	362	278	76.80%	< 1.0 × 10^-16^

We calculated *p *value by generating random predictions on the ENCODE regions.

**Figure 4:**
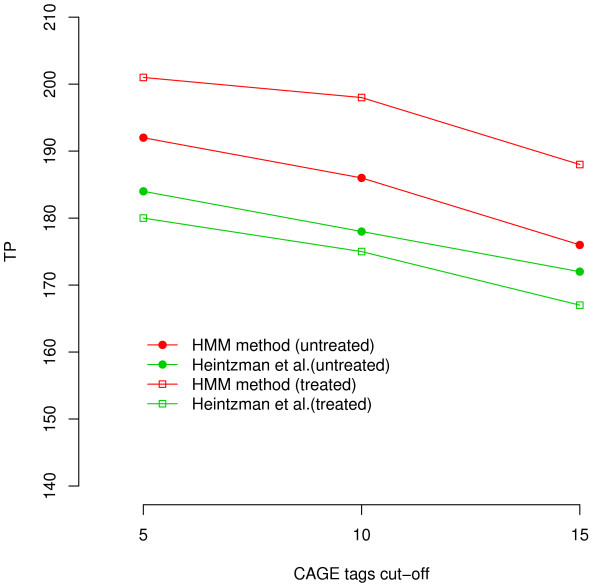
**Promoter predictions supported by CAGE tags**. We compared the number of predicted promoters supported by CAGE tags when changing the minimum number of CAGE tags found within 2.5 kb from the predicted TSSs. We compared the results when the HMM method made same number of predictions as the profile-based method (untreated cells: 198 predicted sites, treated cells: 208 predicted sites).

CAGE tags[20] have been generated to map promoters and we investigated if the predicted TSSs were supported by CAGE tags. Ideally, only one CAGE tag is needed to map a promoter. But due to the noise of generating the tags, confident promoter are usually supported by the overlapping with multiple tags. The larger the number of CAGE tags overlap with the predicted promoters, the more confident the predictions are. We counted the number of predicted promoters supported by at least 5, 10, and 15 CAGE tags. We predicted 198 and 208 promoters in the untreated and the treated cells, respectively, for both the HMM and the profile-based method (Figure 4). When the CAGE tag cutoff was 5, the HMM method found 192 (PPV = 96.97%) and 201 (PPV = 96.63%) promoters supported by CAGE tags in the untreated and the treated cell, respectively, compared with 184 (92.9%) and 180 (86.5%) supported predictions by the profile-based method, respectively. It is not surprising that the number of the supported promoters decreased when we increased the minimum number of overlapping CAGE tags. We found that the performance improvement of the HMM over the profile-based method was more significant in the treated cells. Considering both methods were trained using the untreated data, it suggests that the HMM method is more robust than the previous method.

We next compared the performance of the two methods on predicting active promoters. Gene expression measurements[21] showed that there were 177 active and 155 inactive promoters in the untreated HeLa cells, and 181 active and 151 inactive promoters in the treated cells. The profile based method detected 127 active and 31 inactive promoters in the untreated cells (Table 5). The two methods correctly predicted similar number of active promoters (127 and 128) when the number of predictions was around 200. While increasing the number of predictions, the profile based method did not but the HMM method did make more correct predictions of active promoters in both treated and untreated cells (Table 5).

**Table 5:** Comparison of active promoter predictions.

untreated cell^a^				
	Active promoters			Inactive promoters

	Total Prediction	Expression Supported Prediction	PPV	

Heintzman *et al. *[15]^b^	197	127	64.47%	31

	229	127	55.46%	32

HMM (*c*_1 _= 1.95)	197	128	64.97%	25

HMM (*c*_1 _= 1.6)	229	135	58.95%	31

HMM (*c*_1 _= 0.5)	309	143	46.28%	40

treated cell^a^				

	Active promoters			Inactive promoters

	Total Prediction	Expression Supported Prediction	PPV	

Heintzman *et al. *[15]	204	128	62.75%	23

	213	128	60.09%	23

HMM (*c*_1 _= 1.853)	204	128	62.75%	19

HMM (*c*_1 _= 1.367)	247	139	56.27%	22

HMM (*c*_1 _= 0.5)	328	145	44.21%	30

^a^The total numbers of predictions in Table 5 are slightly different from Table 4 because when multiple predicted sites were supported by the same TSS or any enhancer evidence, we merged these predictions (see Methods).

^b^The number of correctly predicted active promoters did not change using a lower cut-off in the profile-based method.

**Figure 5:**
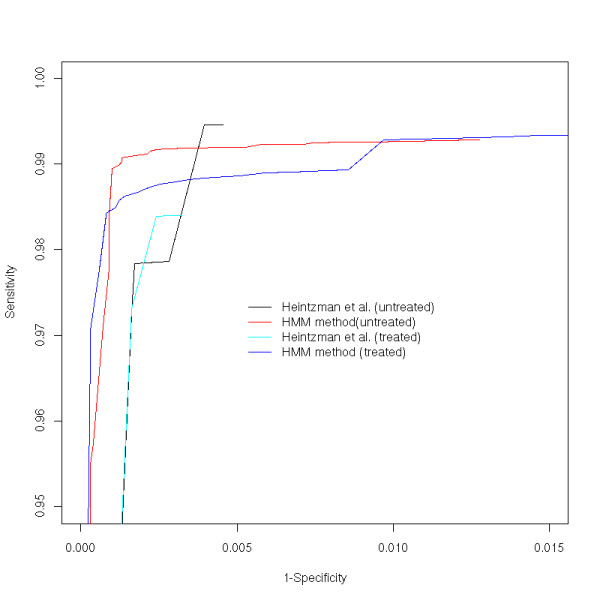
**ROC curves of the HMM and profile-based methods in the untreated and treated cells**.

We investigated the sensitivity and specificity of each method (for the definition see Methods). Figure 5 shows the receiver operator characteristic (ROC) curves of the HMM and the profile-based methods in the untreated and treated cells. Both methods achieved good performance with high sensitivity and specificity but the performance improvement of the HMM method is prominent.

### Enhancer prediction in the ENCODE regions

We also used the trained HMM to predict enhancers in the ENCODE regions. We found 319 (82.01%) and 243 (75.00%) common enhancers predicted in the untreated (389 predictions) and treated cells (324 predictions), respectively, by the HMM method and the profile-based method with the same number of total predictions. To compare the performances of the two methods, we checked how many of them were supported (within 2.5 kb) by nearby p300 and TRAP200 binding sites as well as DNase hypersensitivity sites (DHSs). p300 is a transcriptional co-activator[22,23]. TRAP220 is a component of the Mediator complex[22,23] that have been shown to bind to enhancers as well as promoters. DHSs are nucleosome free regions that are often occupied by enhancers [24]. We only considered p300, TRAP220 and DHS sites that are distal (> 2.5 kb) from any TSS to avoid confusion with promoters.

**Figure 6:**
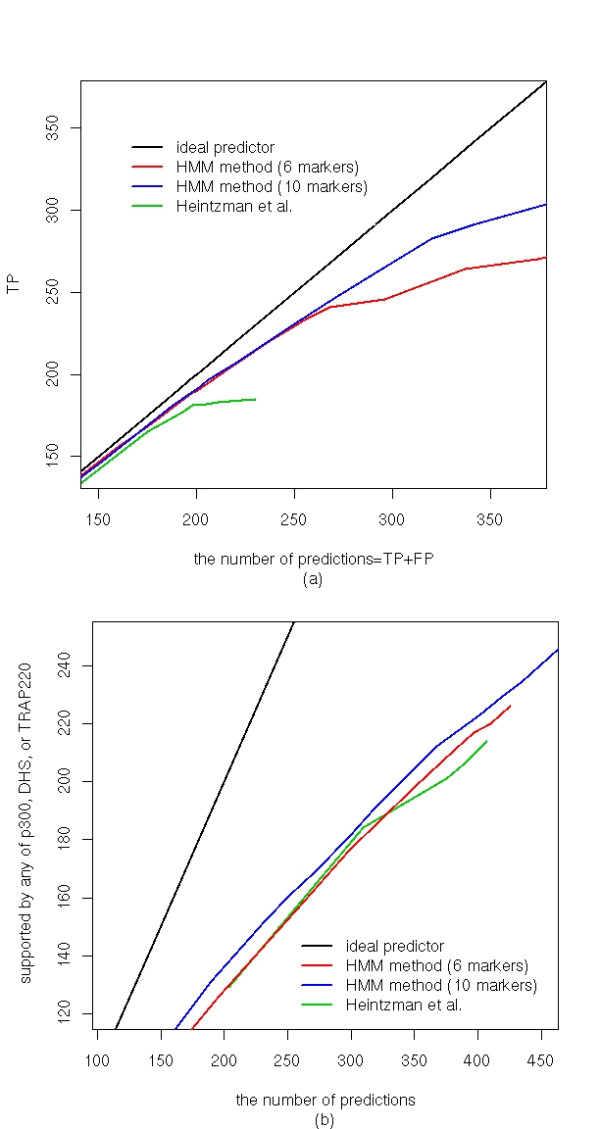
**Comparison of (a) promoter and (b) enhancer prediction**. The prediction results using 10 histone marks are compared with those using 6 histone marks.

In the untreated HeLa cells, all three sites have been mapped in the ENCODE regions. We calculated sensitivity = TP/(TP+FN) of the two methods (Table 6). More predictions by the HMM method were supported by any and all of the three lines of evidences. In total, 213 out of 389 predictions by the HMM method were supported (PPV = 54.76%) by any of the three evidences, while the profile-based method made 206/389 = 52.96% supported predictions (Table 6 and Figure 6b). We should point out that there may exist true enhancers among the predicted ones by HMM but not supported by the p300, DHS or TRAP220 data. This may explain the relatively smaller improvement of our method over the profile-based method on enhancer predictions than on promoter predictions.

**Table 6:** Comparison of enhancer predictions in the untreated Hela cells.

	Heintzman *et al*. [15] total 389 prediction	HMM method total 389 prediction
distal p300 (n = 94)	77 (sensitivity = 81.91%)	82 (sensitivity = 87.23%)

distal DHS (n = 587)	165 (sensitivity = 28.11%)	179 (sensitivity = 30.49%)

Distal TRAP220 (n = 77)	43 (sensitivity = 55.84%)	47 (sensitivity = 61.04%)

Any of distal (DHS, p300, TRAP220)	206 (PPV = 52.96%)	213 (PPV = 54.76%)

Sensitivity = (TP/(TP+FN)) or PPV = (TP/(TP+FP)) was calculated.

In the treated cell, only p300 binding data was available and it was used to evaluate the predictions of the two methods. While Heintzman *et al*. had 104 out of 318 predictions overlapping with p300 sites (sensitivity = 104/147 = 70.75%, PPV = 32.70%), the HMM method found 109, out of 288, p300-supported predictions (sensitivity = 76.22%, PPV = 37.85%). Again, the HMM method outperformed the profile-based method in this test set.

### Including additional histone marks can further improve the performance of the HMM method

Recently, Hon et al. conducted the same ChIP-chip experiments on more histone modification marks, H3K9Ac, H3K18Ac, H3K27Me3 and H3K27Ac, in the ENCODE regions[25]. A robust method should achieve better performance when including additional data. We applied the HMM method to this larger dataset and evaluated its performance as above. After training the HMM predictor using all ten histone marks and a window size of 2 kb, same as in the six histone mark dataset, we predicted promoters and enhancers in the ENCODE regions. We observed a significant improvement in the promoter predictions (Figure 6a). The HMM method using 10 histone marks was quite close to the ideal line even when other methods reached plateau. For example, the HMM made 291, out of 341, correct predictions (PPV = 291/341 = 85.34%) using 10 histone marks and only 264 out of 337 correct predictions using 6 histone makers. Such improvement became more significant when more predictions were made.

The performance of the HMM method on enhancer prediction was also improved using more histone marks (Figure 6b). For example, 232 enhancers were correctly predicted (PPV = 54.46%) using the 10 histone marks, compared with 226 correct predictions (53.05%) among the same number (426) of the total predictions using the 6 histone marks. The improvement was not as significant as in the case of the promoters. It is possibly because the evidences of true enhancers (p300/TRAP200 binding and DHS sites) are not as direct as those for the promoters (the annotated TSSs were determined using full length cDNA).

### Prediction of active and inactive promoters using genome-wide ChIP-Seq data

Compared with ChIP-chip, ChIP-Seq is more costly effective and probably also less noisy on mapping chromatin modifications at the genome-wide scale. We investigated how well our method works with the ChIP-Seq data generated by Mikkelsen et al. in the three mouse cell lines[8]: embryonic stem (ES) cells, neural progenitor cells (NPCs) and embryonic fibroblasts (MEFs). We first compared the patterns of the four histone marks, H3K4me3, H9K4me3, H3K27me3, and H3K36me3, around TSSs because these four marks were measured in all the three cell lines. We assigned each promoter to one of the four groups based on the gene expression level measured in the same study[8]. We averaged the sequencing read counts of each group around TSS. The active and inactive promoters exhibit distinct patterns of all but H3K9me3 marks (Figure S2, see Additional file 7). Strong signals of H3K4me3 in the active promoters and H3K27me3 in the inactive promoters are consistent with their known functions. H3K36me3, a mark for transcriptional elongation, shows a quite spread out pattern around TSS.

Next, we trained two HMMs on 200 active and 200 inactive promoters randomly selected from the ES cell and predicted the promoters in all three cell lines. Because the active promoters contain stronger chromatin modification signals (more sequencing reads) than the inactive promoters, our method predicted more active promoters than inactive ones. The majority of the predicted promoters were within 2.5 kb of the annotated RefSeq TSSs (Table 7): > 81% for active promoters and > 66% for inactive promoters. For the predictions located more than 2b from the annotated TSSs, these sites can be unannotated promoters or false positives. We then assessed the prediction accuracy of our model using gene expression. Among the genes that could be unambiguously called active or inactive, our method correctly predicted the activity of the majority of the promoters. Considering the scale of our predictions, the PPVs of both active and inactive promoter predictions (expression supported) are satisfactory: > 88% and ≥ 74%, respectively, for the two classes.

**Table 7:** Predicted active and inactive promoters in the mouse genome.

Active Promoter^a^					
Cell lines	Active Gene	Total Prediction	Refseq Supported PPV	Predicted promoters not present in the expression measurement	Expression Supported PPV

ES	7887	13853	81.4%	7191	88.6%

MEF	8092	11913	88.1%	5480	92.3%

NPC	7413	12700	84.1%	6259	89.0%

Inactive Promoter^b^					

Cell lines	Inactive Gene	Total Prediction	Refseq Supported PPV	Predicted promoters not present in the expression measurement	Expression Supported PPV

ES	4753	2862	77.0%	1806	79.2%

MEF	4248	4301	66.1%	3061	74.6%

NPC	5259	422	73.2%	267	94.8%

^a ^Active promoter supported by gene expression and Refseq: TP is the number of active genes prediction as active and FP is the number of inactive genes predicted as active. ^b ^Inactive promoter supported by gene expression: TP is the number of inactive genes predicted as inactive and FP is the number of active genes predicted as inactive. In both cases, we only considered predictions located within 2.5 Kb to annotated genes and the total number of predictions is thus usually larger than the sum of TPs and FPs. Refseq supported PPV shows how much percent of the total active/inactive promoter predictions are supported by Refseq.

## Conclusion

We present here an HMM method to predict promoters and enhancers using their characteristic histone modification patterns. We used a HMM-SA procedure to automatically select the most informative and the optimal window size of histone modifications. We showed that the more histone marks are considered, the better the performance of the HMM can achieve. We compared the HMM method with the best prediction results using the profile-based method in the Heintzman et al. study. The cross-validation test showed that the HMM method performed better than the profile-based method, especially in the enhancer classification (Table 3). This observation suggests that the HMM method has a better capability to learn complicated patterns particularly for the weak signals around enhancers. Because correct identification of distal enhancers is critical in deciphering transcriptional regulation, this feature of HMM gives it an edge over the profile-based method.

We also found that the window size of 2 kb gave the best balance between inclusion of sufficiently strong signals and exclusion of non-informative ones that undermine the prediction accuracy. However, the improvement of using a 2 kb window instead of 10 kb was rather small compared to the use of HMM (Table 1). It suggests that the improvement in classification is mainly from the HMM's ability to capture the characteristic patterns of histone modifications for multiple marks.

We demons trated that the HMM method outperforms the previously developed profile-based method on predicting promoters and enhancers using chromatin signatures, particularly on the independent test dataset in the HeLa cells treated with IFN*γ*. The profile-based method performed well with small number of predictions. It reached the maximum true positives (TPs) when the number of promoter predictions was about 230 (Table 4 and Figure 6). Beyond 230, TPs almost do not increase with the number of predictions. In contrast, the HMM method keeps making correct predictions and it outperformed the profile-based method even more significantly (Figure 6). The improvement in enhancer prediction is not significant (Figure 6), which may be due to the limited knowledge of enhancer positions in the genome. We only evaluated the prediction accuracy using the DHS and the binding sites of p300 and TRAP220 that may miss many enhancers.

The HMM method is also less sensitive to noise in individual histone modifications. As shown in Figure 2(A) the profile method failed to find a TSS where H3K3me3 signal is weak. The HMM method predicted this TSS by using all the histone marks. In Figure 2(B) the HMM method predicted an enhancer that is supported by both p300 and DHS sites. Weak signal of H3K4me3 may cause the failure of the profile based method of identifying this site. An opposite example is shown in Figure 2(C) where a relatively stronger H3K4me3 signal than typical enhancers prevents identification of DHS site to be enhancers by the profile-based method while the HMM method was not affected.

In the present work, we did not further distinguish sub-clusters of promoters and enhancers as in the study of Heintzman *et al*. to avoid overfitting. It is very likely that promoter and enhancers may have distinct histone modification patterns depending upon their functional state (active, repressed or poised) [26]. As histone modification data are becoming available on more histone marks and on the entire human genome [6], it is possible to train separate or refined HMMs for promoter/enhancer in different functional states, which should further improve the performance of our model.

We also demonstrated the success of our approach on analyzing ChIP-Seq data. By including chromatin marks that are characteristics of transcription, our method could successfully predicted the activities of promoters. If annotated enhancers are available for training the HMMs, it is straightforward to extend our predictions to enhancers. With the fast accumulation of chromatin modification data, we believe that our method will provide a useful tool in systematically mapping regulatory elements.

## Methods

### Data Preparation

The histone modification data were obtained from the Heintzman *et al. *study [15]. The averaged profile and individual histone marks are shown in Figure 1, comparing the histone patterns on promoter and enhancer. We followed their smoothing procedure. Data were grouped into 100 bp bins and the values of probes within each bin were averaged, *e.g*. a histone pattern of 2 Kb consists of 20 bins. The regions not covered by probes were linearly interpolated if the size of the uncovered region is less than 1000 bp. Heintzman *et al. *studied histone modifications in both untreated HeLa cells and HeLa cells treated with IFN*γ*. To design a classifier, HMMs were trained on promoters, enhancers and background, respectively. Previous studies demonstrated that p300 and related acetyltransferases are present at enhancers and promoters[23]. Heintzman *et al. *determined 124 and 182 p300 binding sites in the untreated and treated HeLa cells, respectively. We used 74 p300 binding sites in the untreated cells after removing those within 2.5 kb of the known 5' ends of genes. These sites were enriched with DNaseI hypersensitive sites (69.7%) and over 60% of them were conserved across species [15]. These evidences strongly support that distal p300 binding sites represent a subset of enhancers. Heintzman *et al. *used 106 active promoters in the untreated cells that were centered at annotated RefSeq TSSs as their training data for promoters [15]. In the current study, one promoter and one enhancer were deleted from the training set used by Heintzman *et al. *because they included many unprobed regions.

While Heintzman *et al. *only tested on the window size of 10 kb centered on TSS and p300 binding sites, we tested various window size. The candidate window sizes of histone marks for the HMM-SA procedure were 1, 2, 4, 6, 8, 10, and 12 kb. Once the optimal window size 2 kb was selected by HMM-SA, all the training dataset of 105 promoters and 73 enhancers were used to train HMMs to predict promoters and enhancers in the ENCODE regions. The histone patterns in the cell treated with IFN*γ *were used as an independent test set.

### The HMM classifier

**Figure 7:**
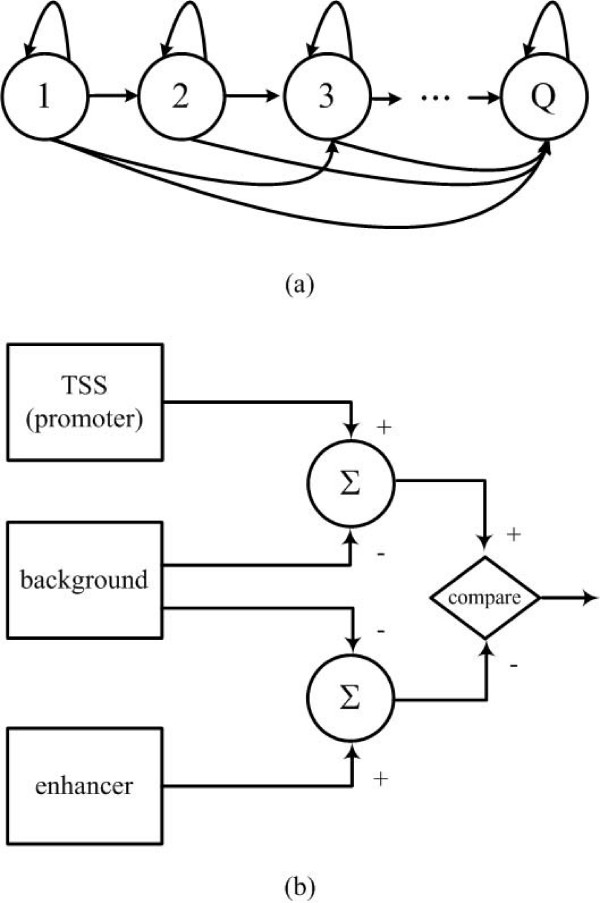
**The HMM Classifier**. (a) A left-right HMM with *Q *states. Each state has a transition to itself and outgoing transitions toward higher states behind. Once a state is left it never comes back in a left-right model. (b) Three HMMs are trained separately for promoter, enhancer and background. Log-odds are calculated to classify a genomic region (see Methods).

We designed an HMM(Θ) with left-right structure[17] to represent the histone modification patterns. Left-right structure has been widely used in speech recognition to capture signal pattern, which serves well for our purpose of capturing histone modification patterns. The HMM has *Q *states (Figure 7). An HMM state emits a signal according to a probability density function of mixture Gaussian of *N *dimension. Here *N *is the number of histone modification patterns under consideration. The probability density function of the mixture Gaussian is

$$\begin{array}{cc} b_{j}(x)=\sum_{m=1}^{M}c_{jm}G[x,\mu_{jm},U_{jm}], & 1\leq j\leq Q \end{array}$$

where ***x ***is the vector being modeled and *c*_*jm *_is the mixture coefficient for the *m*th Gaussian in state *j*; *G *[**x**, ***μ***_*jm*_, **U**_*jm*_] represents the Gaussian function with mean vector ***μ***_*jm *_and covariance matrix ***U***_*jm*_. The forward and backward algorithm[17] was used to estimate the transition probabilities and the mixture coefficients in each state. We trained three HMMs for promoters, enhancers and background separately. We set *Q = (number of bins)/k *to change the number of states depending on the length of data (we set *k *= 8) and the minimum *Q *was set to 3. The background HMM was designed to have the minimum number of states (*Q *= 3). Each state is composed of 3 mixtures of Gaussian components (*M *= 3) to capture the complex histone modification patterns. Models with larger *m *did not improve the prediction performance (data not shown).

For a given genomic region, a log likelihood score was calculated using the three HMMs for promoter, enhancer and background. Two log-odd scores were calculated as the following.

(1) $$\text{log-odd for promoter}:\log\frac{P\text{(}x|\Theta^{promoter})}{P(x|\Theta^{background})}>c_{1},$$

(2) $$\text{log-odd for enhancer}:\log\frac{P\text{(}x|\Theta^{enhancer})}{P(x|\Theta^{background})}>c_{2}$$

The log-odd score reflects how strong a signal is compared to the background. If the log-odd is below a cutoff (*c*_1_, *c*_2_), it is regarded as a background signal. The number of prediction depends on these cut-off values. We plotted Figure 3 and 6 while changing the cut-off values.

When we scan the ENCODE regions, we smoothed results by averaging adjacent 3 log-odds and took peaks of the log-odds of promoter over enhancer. This smoothing procedure reduced fluctuations of log-odds along the chromosome, especially at the boundaries of the unprobed regions. If multiple predictions were made within 1.5 Kbp, only the prediction with the highest log-odds was kept. If a promoter and an enhancer were predicted within 1.5 Kb, we only kept the prediction with the higher log-odd. We examined the percentage of promoters and enhancers being correctly predicted while varying the cutoff values *c*_1 _and *c*_2 _(Figure 3, Figure 6 and Table 4). Using six histone marks we observed the same number of prediction of the HMM predictor as the profile-based method when *c*_1 _= 2.205 (untreated) and *c*_1 _= 2.1 (treated). We used *c*_2 _= 0.25 (untreated) and *c*_2 _= 0.0 (treated) to compare the prediction result of the enhancer (Table 6).

### Search for the most informative histone modification combination

Automatic search for the most informative combination of histone modification is a typical feature selection problem. We took an approach that couples HMM with simulated annealing (SA) to find the optimal combination. SA [16] is a generic approach for global optimization problems. Incorporating a temperature parameter into the optimization procedure, it explores broad parameter space at high temperatures and restricts exploration at lower temperatures. The simulated annealing updates were made based on the Metropolis criterion. The Metropolis criterion makes a change from *E*_*current *_to *E *with a probability of

(3) $$\min\left(1,\exp\left\{-\frac{E-E_{current}}{T}\right\}\right)$$

That is, if *E*_*current *_is greater than the previous value (*E*), the move is always accepted; otherwise, the move is accepted with a probability of $\exp\left\{-\frac{E-E_{current}}{T}\right\}$ that decreases with *T*.

To adapt the SA method to our model, we hybridized HMMs with SA. Initially, SA randomly selected a candidate combination of histone modifications. Also, a window size was randomly selected among 1, 2, 4, 6, 8, 10, 12 Kbp. An HMM was trained with the candidate combination and evaluated by *E*_*current*_. *E*_*current *_is defined as:

(4) *E*_*current *_= (sensitivity of promoter × 100) + (sensitivity of enhancer × 100).

The combination is accepted with the probability given in equation (3). *E*_*current *_is always accepted if *E*_*current*_>*E*; otherwise, it is accepted with a probability that generally decreases as the temperature (*T*) decreases. The next move is made by randomly adding or removing one or two histone patterns and increasing or decreasing one 2 Kb of the window size. This procedure is repeated while decreasing the temperature *T*. In the simulation we used

(5) *T *= 0.9^*iteration*^

In the HMM-SA procedure, the 105 promoter and 73 enhancers in the training dataset was divided into training and evaluation sets, half of them were used to train the HMMs and the other half to calculate *E*_*current*_. The training set (52 promoters and 36 enhancers) and the test set are fixed for each run. We set the maximum number of iterations to be 200 to give SA enough burning period. In fact, most simulations were converged in less than 100 iterations. We recorded the results for 30 independent simulations.

### Evaluating predictions

We validated the prediction results in the ENCODE regions by calculating how many predicted promoters are supported by annotated TSSs in RefSeq. The adjacent 3 log-odds (1, 2) are averaged. If multiple peaks of promoters or enhancers are found within 1.5 kb, only the highest log-odd is selected. A prediction was considered as correct if the predicted center is within *D *= 2.5 kb to the closest annotated TSS of a gene. When multiple predicted sites are supported by the same TSS or any enhancer evidence, we merge these predictions. However, when multiple predicted sites are not within the distance, we counted all of them as FPs. The total number of the predicted promoters in Heintzman *et al*[15] was 208. Since two promoters are referred to the same gene, we treated these two promoters as one and thus the total number of predictions becomes 207. We defined PPV = TP/(TP+FP).

To compare the performance of the two methods, we plotted ROC curves for promoter predictions in both untreated and treated cells (Figure 5). We defined FN as the number of active promoters that were missed in our predictions. It is not very straightforward to define true negatives. We chose to divide the entire ENCODE regions into 2.5 kb-long non-overlapping segments. There were 9928 segments in which no annotated TSSs were found within ± 2.5 kb. We defined TN as the number of segments that did not contain any predicted promoters. The sensitivity and the specificity were given as TP/(TP+FN) and TN/(FP+TN), respectively.

### ChIP-Seq data in the three mouse cell lines

Mikkelsen et al. generated the genome-wide mapping of chromatin modifications in three mouse cell lines: embryonic stem (ES) cells, neural progenitor cells (NPCs) and embryonic fibroblasts (MEFs) [8]. Four chromatin marks, H3K4me3, H9K4me3, H3K27me3, and H3K36me3, were measured in all these cell lines. We trained a HMM classifier using the chromatin modification patterns around TSS in the ES cells and tested it in all three cell lines. Based on the gene expression measured by Mikkelsen et al., we randomly selected 200 active and 200 inactive promoters in the ES cells as the training set. Because there were only four chromatin marks, we used all of them in the HMM model. Similar to analysis of ChIP-chip data, we first used a 2 Kb window to locate TSSs in the genome (see above). Considering the spread out pattern of H3K36me3 that distinguishes active from inactive promoters (Figure S2, see Additional file 7), we next used a 10 Kb window to classify the predicted promoters into active or inactive category. A background HMM was trained using the sequencing reads mapped to chromosome 1.

We evaluated the classification performance of our method using gene expression and RefSeq annotation on predictions that could be unambiguously assigned to a gene, namely located 2.5 Kb within an annotated TSS. Mikkelsen et. al conducted replicate measurements of gene expression in the same cell lines (GEO accession number is GSE8024). There were 13482 unique genes in their experiments. The numbers of active and inactive genes in each cell line were counted using the majority rule in the replicate experiments and the genes with marginal expression levels or conflicting calls were excluded (Table 7).

## Abbreviations

HMM: Hidden Markov Model; TSS: Transcription Start Site; SA: Simulated Annealing; PPV: Positive Predictive Value; TP: True Positive; FP: False Positive; FN: False Negative; TN: True Negative; DHS: DNaseI hypersensitive Site; ROC: Receiver Operator Characteristics; TF: Transcription Factor

## Competing interests

The authors declare that they have no competing interests.

## Authors' contributions

KJW implemented the algorithms, performed all tests, and made all images. IC implemented HMM algorithm. BR and WW conceived of the algorithm and participated in its design and coordination. KJW, IC, BR, and WW wrote the manuscript. All authors read and approved the final manuscript.

## Supplementary Material

Additional file 1 **Figure S1.** Probability density of Gaussian mixtures for the three HMM states trained on promoter and enhancer for each chromatin marker. The x-axis is the log ratio of ChIP-chip intensity. The black curve is the mixture of 3 Gaussian (red curves represent individual Gaussians. (A) H4ac. Analysis on the trained HMM for H4ac. Click here for file

Additional file 2 **Figure S1 (B).** H3ac. Analysis on the trained HMM for H3ac. Click here for file

Additional file 3 **Figure S1 (C).** H3K4me1. Analysis on the trained HMM for H3K4me1. Click here for file

Additional file 4 **Figure S1 (D).** H3K4me2. Analysis on the trained HMM for H3K4me2. Click here for file

Additional file 5 **Figure S1 (E).** H3K4me3. Analysis on the trained HMM for H3K4me3. Click here for file

Additional file 6 **Figure S1 (F).** H3. Analysis on the trained HMM for H3. Click here for file

Additional file 7 **Figure S2 Active and inactive profile of the ChIP-Seq data. **Tag counts at TSS are clustered considering the expression ratio. Histone profiles for active and inactive TSS. Click here for file
